# Comparison of Piezoelectric and High‐Speed Drills for Frontal Beak Osteotomy in Endoscopic Sinus Surgery—Exploratory Study

**DOI:** 10.1111/coa.70081

**Published:** 2026-01-02

**Authors:** Łukasz Skrzypiec, Kornel Szczygielski, Agnieszka Brociek‐Piłczyńska, Dariusz Jurkiewicz, Marta Aleksandra Kwiatkowska

**Affiliations:** ^1^ Department of Otolaryngology and Oncological Laryngology With Clinical Department of Cranio‐Maxillo‐Facial Surgery Military Institute of Medicine—National Research Institute Warsaw Poland

**Keywords:** chronic rhinosinusitis, endoscopic sinus surgery, frontal beak reduction, frontal sinus, high‐speed drill, mucosal healing, piezoelectric surgery

## Abstract

**Introduction:**

Frontal beak reduction is a critical and challenging step in endoscopic sinus surgery (ESS) for chronic rhinosinusitis (CRS). Traditional high‐speed drills can risk thermal injury and soft tissue trauma, whereas piezoelectric surgery uses ultrasonic vibrations for selective bone cutting and may improve surgical precision. This study compared piezoelectric devices with conventional drills for frontal beaks reduction.

**Methods:**

A double‐blind exploratory study with randomisation was conducted during the years 2019–2022. Adult CRS patients (*n* = 43) with prominent nasofrontal beak undergoing ESS were randomised to piezoelectric knife (*n* = 22) or high‐speed drill (*n* = 21). Exclusion criteria included prior sinus surgery, septoplasty, neoplasms or systemic contraindications. Outcomes included Lund–Kennedy, Lund–Mackay, Sino‐Nasal Outcomes Test (SNOT‐22), Visual Analogue Scale (VAS) scores and frontal sinus ostium dilatation, measured pre‐operatively and at 1, 4 and 24 weeks post‐operatively.

**Results:**

Both groups showed significant post‐operative improvements in Lund–Mackay and SNOT‐22 scores (*p* < 0.05). At 1 week, the piezo group had significantly lower Lund–Kennedy scores (*p* = 0.022), suggesting better mucosal healing. Correlations in the piezo group were observed between ostium dilatation and improved Lund–Mackay (*R* = −0.527; *p* = 0.008) and SNOT‐22 (*R* = −0.405; *p* = 0.049) at 24 weeks. No significant differences were found in ostium width gain, VAS scores or complications. With piezo devices, the median time of surgery was increased.

**Conclusion:**

Piezoelectric surgery is a safe, effective alternative to drills for frontal beak reduction in ESS, with potential benefits for early mucosal healing. Further studies are warranted to confirm long‐term advantages.

## Key Points

- Piezoelectric instruments enable selective bone cutting while preserving mucosa and soft tissues, whereas high‐speed drills carry higher risks of thermal injury and imprecise cuts.
- Patients operated on with piezo devices showed faster mucosal healing, reduced oedema and lower Lund–Kennedy scores 1 week after surgery compared to drill use.
- Both groups reported significant symptom relief (SNOT‐22 and VAS), but piezo group patients demonstrated stronger correlations between ostium dilatation and long‐term quality of life improvement.
- At 24 weeks, both methods provided comparable outcomes in radiologic disease resolution (Lund–Mackay scores) and endoscopic mucosal healing, showing no significant long‐term differences.
- Piezoelectric devices offer early recovery benefits and improved surgical safety but come with higher upfront costs, which may be justified in high‐volume centres prioritising patient comfort and reduced post‐operative morbidity.

## 1 Introduction

Chronic rhinosinusitis (CRS) is a prevalent and multifactorial inflammatory condition of the paranasal sinuses, affecting approximately 11% of the adult population globally, with a significant impact on quality of life (QoL) and healthcare utilisation [1, 2, 3]. The disease is now recognised to comprise distinct phenotypes with varying pathophysiology, including CRS with nasal polyps (CRSwNP) and without (CRSsNP), which influences both clinical presentation and therapeutic approach [1]. Despite advances in medical management, many patients with CRS require surgical intervention, most commonly via endoscopic sinus surgery (ESS), to restore sinus ventilation and drainage [4, 5].

One of the most technically challenging aspects of ESS, particularly in revision or recalcitrant cases, is the management of the frontal recess and frontal beak. Precise and atraumatic bone removal in this region is crucial to ensure long‐term patency while minimising complications such as mucosal damage, cerebrospinal fluid leaks and scarring [6, 7]. Traditional high‐speed drills have long been utilised for their efficiency. However, they are associated with considerable risks, including thermal injury and reduced tactile control, particularly in narrow anatomic corridors.

Piezoelectric surgery, a technique that utilises ultrasonic microvibrations to cut mineralised tissues while sparing soft tissues, has emerged as a promising alternative in various fields of craniofacial surgery [8, 9]. Its potential benefits include reduced intraoperative bleeding, preservation of surrounding mucosa and neurovascular structures and enhanced post‐operative healing [10]. In ESS, early reports have demonstrated the feasibility and safety of piezoelectric tools in bony work, including frontal sinusotomies [11, 12].

Piezoelectric devices' microvibrations and irrigation's cooling resulted in precise, atraumatic osteotomies with reduced heat and bleeding, shown to be advantageous in dental, craniofacial and sinus surgery [13].

Despite these promising results, comparative evidence specifically evaluating piezoelectric versus high‐speed drills for frontal beak reduction remains limited. This study aims to evaluate the clinical efficacy, safety and precision of piezosurgery in frontal sinus interventions relative to standard high‐speed drill techniques.

## 2 Material and Methods

### 2.1 Study Design

This double‐blinded exploratory study with randomisation was conducted between the Years 2019 and 2022 at a tertiary referral hospital centre and involved patients with a prominent nasofrontal beak and an indication for ESS due to CRS recalcitrant to nasal steroids. The STROBE checklist was applied to the study design.

Patient allocation was performed using a computer‐generated randomisation method (http://www.randomizer.org). Participants were randomly assigned to undergo frontal beak osteotomy using either a piezoelectric knife (the ‘piezo’ group) or a conventional high‐speed drill (the ‘drill’ group). The randomisation software automatically determined the surgical technique for each consecutive study number.

Access to the randomisation list was restricted to a single investigator—the operating surgeon, who was also one of the co‐authors of the study. This investigator enrolled patients according to the predetermined allocation and performed all surgical procedures, assisted by an independent surgical team. The operating surgeon was not involved in any pre‐operative or post‐operative assessments.

All patients were blinded to their group assignment. Post‐operative evaluations were carried out by independent physicians who were likewise blinded to the type of osteotomy performed.

Patients underwent detailed otolaryngological examination, including nasal endoscopy and computed tomography (CT) evaluations before the surgical procedure on multiplanar sections. Patients were also subjected to post‐operative follow‐up assessments at the 1st, 4th and 24th weeks.

Surgical outcomes obtained with each of the evaluated methods were compared.

Consecutive adult patients were enrolled in the study when they met the following criteria: CRS diagnosed as per EPOS guidelines [4], with no improvement after medical treatment in the form of topical steroids for 12 weeks with or without antibiotics, and willingness to proceed for ESS.

Exclusion criteria included a simultaneously performed septoplasty, previous sinonasal surgery or trauma involving the sinonasal complex, Sampter's triad (aspirin sensitivity, asthma, sinonasal polyposis), sinonasal neoplastic or obstructive lesions, known ciliary dysfunction, cystic fibrosis, osteoneogenesis or Paget's disease. Additional exclusion criteria were bleeding disorders or anticoagulant therapy, pregnancy and the presence of a severe systemic or neuropsychiatric disorder.

In patients enrolled in the study, the diagnosis, bilateral ethmoid disease status and prominent nasofrontal beak with frontal sinus outflow tract stenosis (Grade 4 according to the Classification of the Extent of Endoscopic Frontal Sinus Surgery [EFSS]) [7] were confirmed via radiological examination of CT. The disease was staged with the Lund–Mackay system [14].

The study protocol was accepted by the Institutional Ethics Committee, protocol number 75/WIM/2017. Written informed consent was obtained from all participants before enrolment.

### 2.2 Surgery

Two types of instruments were used for the osteotomy of the prominent nasofrontal beak: in the ‘drill’ group—conventional rotary instruments using the Straightshot M5 microdebrider, Medtronic (Jacksonville, FL, USA) with application‐specific single‐use burs.

In the ‘piezo’ group—surgical instruments for ultrasound osteotomies Piezotome SOLO M+, Satalec (Merignac, France) using a special single‐use application tip designed for osteotomy (long ultra‐sharp saw BS1 XXL and long scalpel BS6 XXL).

All operations were performed by a single experienced surgeon under general anaesthesia. Patients underwent endoscopic maxillary antrostomy, ethmoidectomy, sphenoidectomy and frontal sinus outflow tract stenosis exposure. Prominent nasofrontal beak osteotomy was performed using one method: in the ‘drill’ group—with rotary high‐speed instruments, and in ‘piezo’ group—with a piezoelectric tool. At the end of each procedure, a Rapid Rhino 4 cm Riemann Nasal Dressing (Smith & Nephew, RR400) was placed in the ethmoidal region. Before insertion, each dressing was soaked in dexamethasone solution (4 mg per dressing). The dressings were removed 7 days post‐operatively.

The duration of the operation was recorded in each case.

After surgery, patients were prescribed an antibiotic (cephazolin 1000 mg intraoperatively and after surgery and 500 mg every 8 h during hospitalisation time and cefuroxime 500 mg every 12 h for 5 days) and a painkiller (acetaminophen 1000 mg every 6 h) for 7 days.

At baseline, clinically relevant data regarding age, gender, history of allergy or asthma and aspirin hypersensitivity were obtained from patients' medical records. Furthermore, data regarding any previous sinonasal surgery or trauma, the presence of severe diseases and chronic topical or systemic therapy were recorded.

The Lund–Kennedy system used for the evaluation of nasal endoscopy assesses five visual pathologies within the nose and paranasal sinuses: polyps, discharge, oedema, scarring and crusting. Each item is graded from 0 to 2 for each side; hence, total scores range from 0 to 10 and higher scores indicate worse observed disease [15].

Lund–Kennedy endoscopic grading was performed before surgery and 1, 4 and 24 weeks after surgery.

The Lund–Mackay score is used to quantify the radiologic severity of the disease based on sinus CT scans. The extent of radiologic disease is graded from 0 to 2 for each paranasal sinus and for the ostiomeatal complex, with total scores ranging from 0 to 24. Higher scores indicate greater disease severity [15].

Evaluation with the Lund–Mackay scoring system was performed before surgery and 24 weeks after.

Measurements were done by two experienced surgeons independently (excluding the operating surgeon) on coronal, axial and sagittal planes of high‐resolution CT scans. The narrowest width of the frontal sinus ostium was measured in millimetres to one decimal place of precision, after which the results were averaged.

### 2.3 Evaluation of QoL and Symptom Severity

The 22‐item Sino‐Nasal Outcomes Test (SNOT‐22) is a tool for the evaluation of health‐related QoL impairment in patients with CRS. This test consists of 22 questions categorised into five main domains: rhinological symptoms, extra‐nasal rhinological symptoms, ear and facial symptoms, sleep dysfunction and psychological disease domain. Each question is rated from 0 (‘*no problem at all*’) to 5 (‘*worst possible symptom*’) points; hence, possible total scores range from 0 to 110 points, with higher total scores indicating worse symptoms [16]. SNOT‐22 scores can be classified as ‘*mild*’ (score 8–20), ‘*moderate*’ (scores > 20–50) and ‘*severe*’ (scores > 50) [17].

The SNOT‐22 test was performed before surgery and 1, 4 and 24 weeks after surgery.

Visual Analogue Scale (VAS; 0–10) was applied for seven CRS symptoms (facial pressure, headache, nasal blockage, watery discharge, purulent discharge, itchiness and epistaxis). Measured immediately post‐operatively on Days 0 and 7.

### 2.4 Statistical Analysis

Statistica 13.0 software was used. Normality was assessed with the Shapiro–Wilk test. Quantitative data were expressed as mean ± SD or median (range), and qualitative data as percentages. Tests used included Student's *t‐*test, Mann–Whitney *U*, Wilcoxon pairs or Friedman ANOVA as appropriate. Correlations were analysed with Pearson's or Spearman's coefficients. Statistical significance was set at *p* < 0.05, with Bonferroni corrections for multiple testing.

## 3 Results

The total number of 43 patients (including 28 males and 21 females) was subjected to the study. A total of 21 patients (49%) had prominent nasofrontal beak removal performed using ‘drill’, and in 22 patients (51%) a piezoelectric knife was used for osteotomy. The detailed description of patients' clinical characteristics and comorbidities is given in Table 1.

**TABLE 1:** Clinical characteristics of the study groups.

Variable	‘Piezo’ group *n* = 22	‘Drill’ group *n* = 21	*p*
Age (years)			
Mean (SD)	48 (SD: 1.41)	48.14 (SD: 19.09)	0.97
Range	26–73	27–73	
CRSwNP, *n* (%)			
Yes	11 (50%)	14 (67%)	0.358
No	11 (50%)	7 (33%)	
Asthma, *n* (%)			
Yes	4 (18%)	8 (38%)	0.185
No	18 (82%)	13 (62%)	
Allergy, *n* (%)			
Yes	6 (27%)	7 (33%)	0.20
No	16 (73%)	14 (67%)	
GERD, *n* (%)			
Yes	2 (10%)	4 (19%)	0.41
No	20 (90%)	17 (81%)	
Diabetes mellitus, *n* (%)			
Yes	0 (0%)	0 (0%)	1.00
No	22 (100%)	21 (100%)	
NSAID treatment, *n* (%)			
Yes	1 (5%)	1 (5%)	1.00
No	21 (95%)	20 (95%)	
Others,^a^ *n* (%)			
Yes	8 (36%)	4 (19%)	0.22
No	14 (64%)	17 (81%)	

Abbreviations: CRSwNP, chronic rhinosinusitis with nasal polyps; GERD, gastroesophageal reflux disease; *n*, number; NSAID, non‐steroidal anti‐inflammatory drug; *p*, level of statistical significance.

^a^ Other diseases and disorders including hypertension, hypothyroidism, hyperthyroidism, cataracts, hearing loss, epilepsy, pituitary macroadenoma and post‐surgery condition.

Statistical comparisons were performed using chi‐square or Fisher's exact test for categorical variables. No significant differences were observed between groups (CRSwNP *p* = 0.358; asthma *p* = 0.185; all other comparisons *p* > 0.05).

The median time of surgery in the ‘piezo’ group was 132.27 min (range: 110–240) and in the ‘drill’ group it was 97.38 min (range: 70–125).

The width of dilatation of the sinus ostium before and after the procedure did not differ regarding surgery type (*p* > 0.05). In the drill group, the mean value of frontal ostium width was 5.15 mm before surgery and 7.55 mm after, whereas in the piezo group, the mean values were 5.02 and 7.99 mm, respectively.

In patients operated on with a piezoelectric knife, a statistically significant correlation was found between the Lund–Mackay score at 24 weeks after surgery and the degree of dilatation of the sinus ostium (Spearman's *R* = −0.527; *p* = 0.008). There was no relationship between Lund–Mackay outcomes and sinus ostium dilatation in patients operated on with a high‐speed drill (*p* > 0.05). The detailed analysis is given in Table 2.

**TABLE 2:** Lund–Mackay score outcomes before and after surgery.

CT Lund–Mackay scores		Before surgery (baseline)	24 weeks after surgery
‘piezo’ group	Median (range)	7 (5–12)	2 (0–7)
‘drill’ group	Median (range)	7 (2–11)	2 (0–8)
*p* value regarding differences between groups at the same visit		0.377	

Sinus ostium dilatation was not related to Lund–Kennedy scores in patients from the ‘piezo’ group, but a moderate negative correlation was found between the percentage of ostium dilatation and improvement assessed by the endoscopic Lund–Kennedy scale 24 weeks after surgery in ‘drill’ group (Spearman's *R* = −0.420; *p* = 0.041).

Concerning whether the type of tool used has an impact on the healing process, statistically significant differences were found at 1 week after surgery—patients from the ‘piezo’ group had a lower Lund–Kennedy scale score than patients from the ‘drill’ group (*p* = 0.022).

**FIGURE 1:**
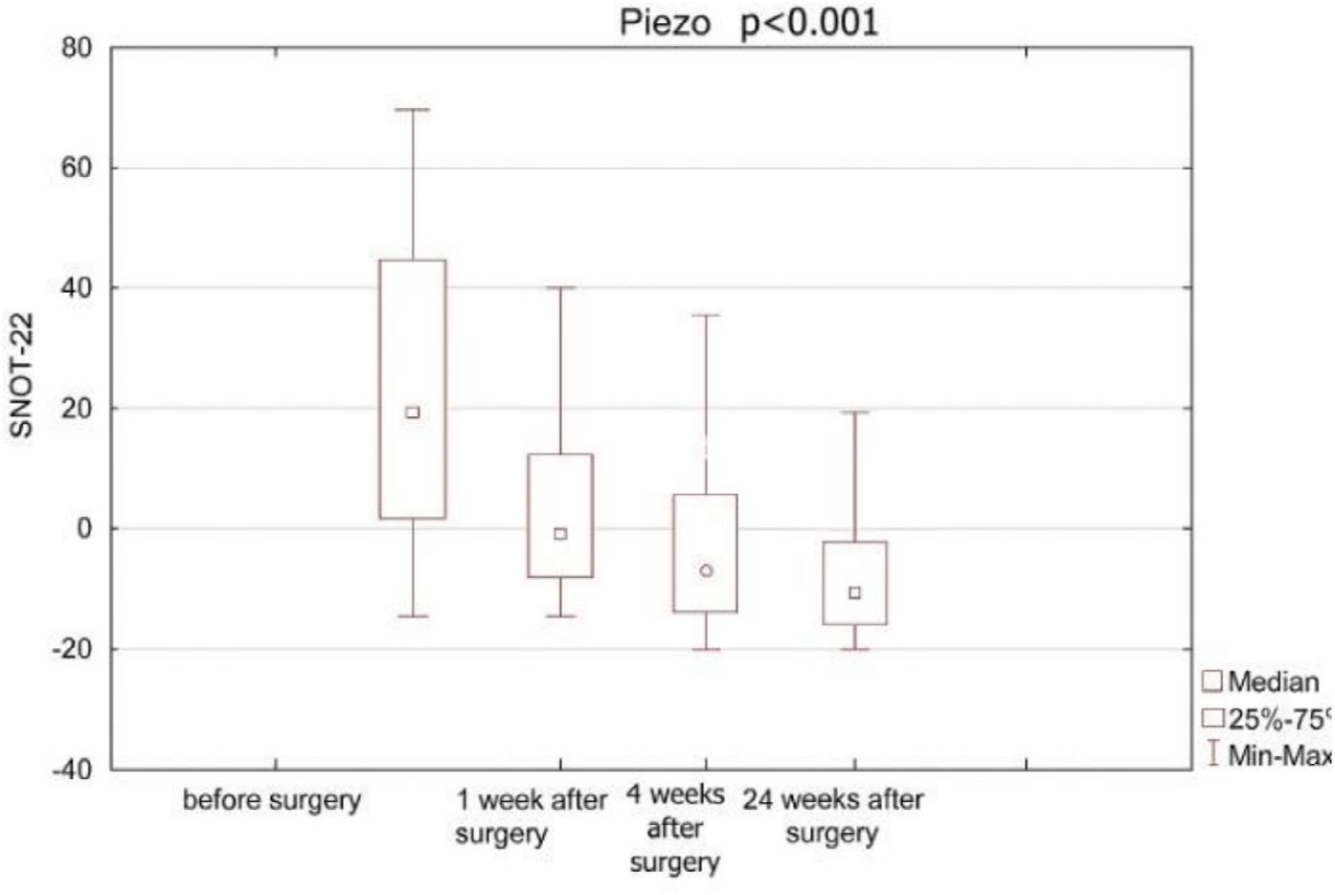
SNOT‐22 outcomes before and after surgery in patients operated with piezoelectric knife.

**FIGURE 2:**
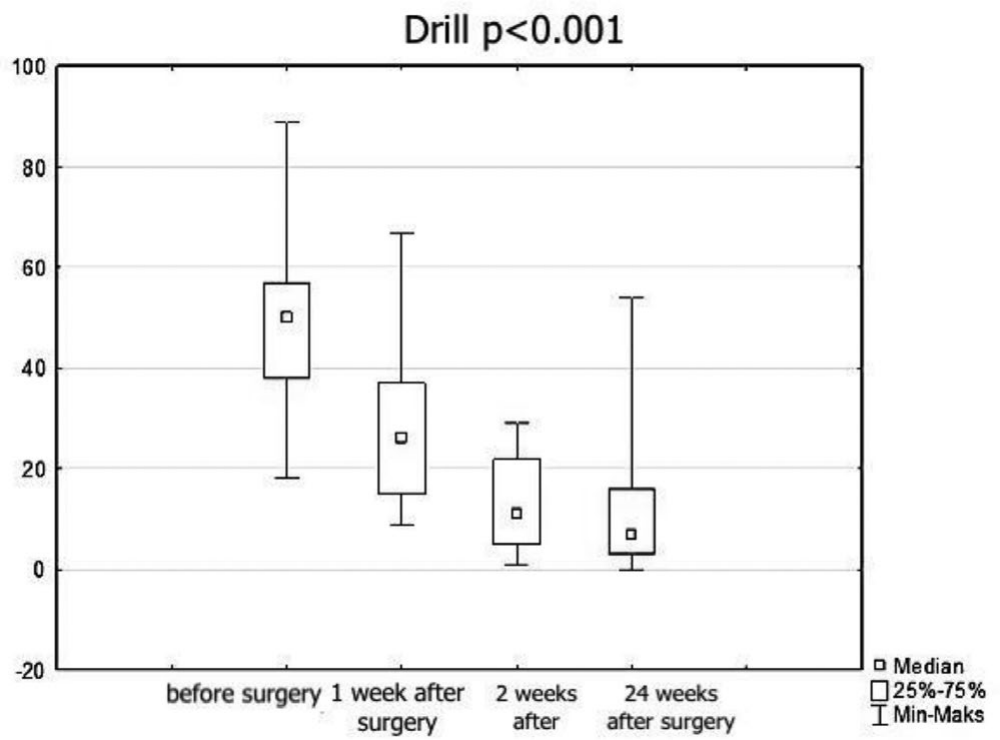
SNOT‐22 outcomes before and after surgery in patients operated on with high‐speed drill.

In the ‘piezo’ group SNOT‐22 scores were statistically significantly lower at each subsequent visit than at the pre‐surgery visit. The comparison of SNOT‐22 results for each of the follow‐up visits is presented in Figures 1 and 2.

Baseline SNOT‐22 scores were compared between the groups to assess potential imbalance. The difference in baseline SNOT‐22 scores between the ‘piezo’ group (mean ± SD: 45.6 ± 22.9) and the ‘drill’ group (49.4 ± 18.2) was not statistically significant (*p* > 0.05, Mann–Whitney *U* test). Therefore, the observed post‐operative improvements were not confounded by baseline symptom severity. Moreover, all analyses of post‐operative outcomes were performed on absolute and relative changes from baseline, minimising the effect of any minor baseline variation.

For patients operated on with the drill, no correlation was found between the SNOT‐22 score and the degree of dilatation of the frontal sinus ostium (*p* > 0.05); however, such a correlation was found in the group of patients operated on with the piezoelectric knife (Spearman's *R* = −0.405, *p* = 0.049).

In both groups, patients' symptoms alleviate in almost all evaluated items, especially in the case of nasal blockage (item VAS‐c). There were no differences between VAS scores regarding the tool used for surgery (*p* > 0.05). The detailed results of each item are given in Table 3.

**TABLE 3:** VAS scale outcomes at 0 and 7 days after surgery.

VAS scale (median of points [range])		Item						
		a	b	c	d	e	f	g
0 days after surgery	‘piezo’ group	3 (0–10)	3 (0–10)	5.5 (2–10)	2 (0–10)	0 (0–6)	0.5 (0–10)	2.5 (0–9)
	‘drill’ group	3 (0–10)	3 (0–10)	5 (0–10)	2 (0–8)	1 (0–7)	1 (0–8)	4 (0–9)
*p* value regarding differences between groups at the same visit		0.306	0.684	0.469	0.774	0.172	0.820	0.270
7 days after surgery	‘piezo’ group	2 (0–10)	2.5 (0–9)	1 (0–9)	1 (0–8)	1 (0–8)	0 (0–8)	2 (0–9)
	‘drill’ group	2 (0–8)	2 (0–6)	2 (0–10)	1 (0–7)	1 (0–6)	1 (0–4)	2 (0–8)
*p* value regarding differences between groups at the same visit		0.729	0.774	0.714	0.804	0.774	0.684	0.670

*Note*: a Facial and ocular pressure; b, headache/pain on the face; c, nasal blockage; d, nasal watery discharge; e, nasal yellow, green or brown discharge; f, itchy nose; g, epistaxis.

For the ‘piezo’ group, no correlations were found on Days 0 or 7, but Lund–Mackay at 24 weeks correlated with VAS nasal blockage (*R* = −0.434; *p* = 0.034) and itchiness (*R* = −0.473; *p* = 0.020).

Moreover, a positive moderate correlation was found between SNOT‐22 assessed 1 week after surgery and the VAS‐e score 1 week after surgery (Spearman's *R* = 0.450, *p* = 0.028).

For the ‘drill’ group, for the VAS scale assessed 7 days after surgery, a significant correlation was found between the baseline Lund–Mackay scale score and items VAS‐c (Spearman's *R* = −0.518; *p* = 0.008) and VAS‐g (Spearman's *R* = −0.528; *p* = 0.007) and between baseline Lund–Kennedy scores and the VAS‐c item (Spearman's *R* = 540; *p* = 0.005). In turn, Lund–Mackay scores assessed 24 weeks after surgery correlated with items VAS‐d (Spearman's *R* = −0.433; *p* = 0.031), VAS‐e (Spearman's *R* = −0.402; *p* = 0.046) and VAS‐g (Spearman's *R* = −0.500; *p* = 0.011) assessed 0 days after surgery and with item VAS‐d (Spearman's *R* = −0.527; *p* = 0.007) assessed 7 days after surgery. Lund–Kennedy scores 1 week after surgery correlated with the VAS‐f item at 0 days after surgery (Spearman's *R* = 470; *p* = 0.018). Moreover, a statistically significant moderate positive correlation was found between SNOT‐22 assessed 1 week after surgery and the VAS‐g at 0 days after surgery (Spearman's *R* = 0.488; *p* = 0.013) as well as between VAS‐c (Spearman's *R* = 0.414; *p* = 0.040), VAS‐e (Spearman's R = 0.444; *p* = 0.026) and VAS‐g (Spearman's *R* = 0.405; *p* = 0.045) assessed 7 days after surgery.

## 4 Discussion

This study aims to directly compare piezoelectric instruments and high‐speed rotary drills in frontal beak reduction during ESS for patients with CRS. By focusing on a critical step of frontal sinus access, presented research seeks to determine whether the theoretical advantages of piezoelectric devices translate into meaningful improvements in surgical and patient outcomes. While both techniques resulted in significant post‐operative improvement in all evaluated parameters, early differences in pain and mucosal healing favoured the piezoelectric group. However, long‐term outcomes, including SNOT‐22, Lund–Kennedy and Lund–Mackay scores, were statistically comparable, confirming that both methods achieved similar efficacy.

To date, studies in orthognathic and maxillofacial surgery have demonstrated reduced post‐operative swelling, improved healing and fewer complications with piezoelectric devices compared to rotary drills and saws. In maxillary sinus lift procedures, piezo surgery has been associated with lower membrane perforation rates, reduced post‐operative pain and faster functional recovery compared to rotary instruments [18].

The initial findings of the presented study suggested that patients in the piezoelectric group experienced a more rapid resolution of early post‐operative symptoms, including pain, swelling and mucosal healing, which is consistent with prior research indicating that piezoelectric instruments cause less thermal and mechanical trauma to surrounding tissues [19, 20].

Mean post‐operative pain scores, assessed using the VAS, showed a trend towards reduced early pain in the piezoelectric group, supporting the hypothesis that minimised tissue trauma correlates with decreased post‐operative discomfort [21]. In addition, early endoscopic assessments showed less mucosal oedema and crusting in the piezoelectric group, which aligns with findings by Kumar et al. that suggest improved mucosal preservation with piezoelectric tools [22].

The operative duration was longer in the piezo group compared with the drill group (mean value of 132.3 vs. 97.3 min, respectively). This difference likely reflects the mechanism of bone removal in piezoelectric surgery, where ultrasonic micro vibrations ‘saw’ through the bone—a process that is inherently slower but offers greater precision and control than high‐speed drilling with a diamond burr. Notably, the selective cutting properties of piezoelectric instruments enable precise removal of mineralised tissue while minimising the risk of soft tissue injury or entrapment in rotating instruments. The increased operative time may also reflect the learning curve associated with the implementation of a new surgical device. Importantly, despite the prolonged duration, no increase in complication rate was observed, supporting the safety and feasibility of the piezoelectric method.

While early post‐operative outcomes favoured the piezoelectric technique in terms of pain reduction and mucosal appearance, these advantages did not persist over time. At 24 weeks post‐operatively, there were no statistically significant differences between groups in SNOT‐22, Lund–Kennedy or Lund–Mackay scores. This indicates that both techniques ultimately achieve comparable disease control, consistent with previous systematic reviews [23]. The radiological assessment using the Lund–Mackay scale demonstrated no significant difference in sinus opacification scores at follow‐up, suggesting similar levels of mucosal healing and disease resolution [14].

Endoscopic examination using the Lund–Kennedy scoring system further supported these findings, with both groups showing comparable scores in mucosal oedema, crusting and polyposis at long‐term follow‐up [15]. These results imply that although piezoelectric devices may offer advantages in early post‐operative comfort, the long‐term structural and functional outcomes are similar.

Cost analysis performed by other authors revealed that piezoelectric devices are generally more expensive upfront due to the cost of the equipment and maintenance [24]. However, when considering the overall healthcare utilisation, including shorter hospital stays and reduced need for post‐operative analgesics, the cost difference may be offset [25]. A detailed cost–benefit analysis showed that in high‐volume centres, the initial investment in piezoelectric technology could be justified by improved patient throughput and satisfaction.

Although previous publications have discussed the higher initial cost of piezoelectric devices [24, 25], our study did not include an economic assessment. Therefore, no conclusions can be drawn regarding cost‐effectiveness based on our data.

Radiologically, the Lund–Mackay scores were used to quantify sinus opacification pre‐ and post‐operatively. The data showed no significant difference in the degree of sinus clearance between the two groups at follow‐up, indicating similar efficacy in restoring sinus aeration. However, subtle differences in mucosal thickening and polyp recurrence were observed on high‐resolution CT scans, with the piezoelectric group demonstrating slightly less mucosal hypertrophy, although these differences were not statistically significant.

Endoscopically, the Lund–Kennedy scale was employed to evaluate mucosal status, crusting and polyposis. Both groups showed significant improvement from baseline, with no statistically significant difference at the final follow‐up. These findings suggest that both techniques are effective in achieving long‐term disease control, with the choice of technique primarily influencing early post‐operative recovery.

In summary, this study indicates that piezoelectric instruments provide long‐term efficacy comparable to rotary drills while offering potential benefits in short‐term post‐operative recovery. These findings support the use of piezoelectric devices as a complementary surgical technique that may improve surgical precision and patient comfort without affecting the overall therapeutic outcome. Future studies involving larger groups and cost‐effectiveness analyses are warranted to clarify the specific role and indications for piezoelectric instruments in frontal sinus surgery.

The main limitation of this study is a relatively small sample and the absence of a prior power calculation, which may limit the generalisability of the findings. Nevertheless, as an exploratory comparative analysis, the study provides data that may influence the design of future randomised controlled trials.

## 5 Conclusions

Piezoelectric instrumentation represents a feasible and safe adjunct to traditional high‐speed drills for frontal beak reduction, particularly in complex anterior skull base anatomy. In this exploratory study, piezoelectric instruments demonstrated some early post‐operative advantages—such as reduced discomfort and favourable short‐term mucosal healing—while long‐term clinical, endoscopic and radiological outcomes were comparable between both techniques. Presented findings support the role of piezoelectric devices as a complementary option that may enhance surgical control and perioperative patient experience in selected cases. Larger, prospective randomised studies, including cost‐effectiveness assessments, are required to more definitively establish the clinical value and optimal indications for piezoelectric instrumentation in EFSS.

## Author Contributions

Conceptualisation: Łukasz Skrzypiec, Dariusz Jurkiewicz and Marta Kwiatkowska. Data curation: Marta Kwiatkowska. Formal analysis: Agnieszka Brociek‐Piłczyńska. Funding acquisition: Kornel Szczygielski. Investigation: Łukasz Skrzypiec. Methodology: Łukasz Skrzypiec and Kornel Szczygielski. Project administration: Dariusz Jurkiewicz. Supervision: Dariusz Jurkiewicz. Validation: Kornel Szczygielski and Agnieszka Brociek‐Piłczyńska. Visualisation: Agnieszka Brociek‐Piłczyńska. Writing – original draft: Łukasz Skrzypiec and Marta Kwiatkowska. Writing – review and editing: Kornel Szczygielski, Agnieszka Brociek‐Piłczyńska, Dariusz Jurkiewicz and Marta Kwiatkowska.

## Funding

This study was conducted with the use of the state‐allocated research funds granted to the Military Institute of Medicine in Warsaw (WIM Grant no. 522).

## Ethics Statement

The study protocol was accepted by the Institutional Ethics Committee with protocol number 75/WIM/2017. Written informed consent was obtained from all participants before enrolment.

## Conflicts of Interest

The authors declare no conflicts of interest.

## Data Availability

The data that support the findings of this study are available from the corresponding author upon reasonable request.

## References

[1] S. H. Cho , D. L. Hamilos , D. H. Han , and T. M. Laidlaw , “Phenotypes of Chronic Rhinosinusitis,” Journal of Allergy and Clinical Immunology: In Practice 8, no. 5 (2020): 1505–1511, 10.1016/j.jaip.2019.12.021. 32389275 PMC7696652

[2] S. Albu , “Chronic Rhinosinusitis—An Update on Epidemiology, Pathogenesis and Management,” Journal of Clinical Medicine 9, no. 7 (2020): 2285, 10.3390/jcm9072285. 32708447 PMC7408732

[3] J. N. Palmer , J. C. Messina , R. Biletch , K. Grosel , and R. A. Mahmoud , “A Cross‐Sectional, Population‐Based Survey of U.S. Adults With Symptoms of Chronic Rhinosinusitis,” Allergy and Asthma Proceedings 40, no. 1 (2019): 48–56, 10.2500/aap.2019.40.4182. 30582496

[4] W. J. Fokkens , V. J. Lund , C. Hopkins , et al., “European Position Paper on Rhinosinusitis and Nasal Polyps 2020,” Rhinology 58, no. S29 (2020): 1–464, 10.4193/Rhin20.600. 32077450

[5] R. R. Orlandi , T. T. Kingdom , P. H. Hwang , et al., “International Consensus Statement on Allergy and Rhinology: Rhinosinusitis,” International Forum of Allergy & Rhinology 6 (2016): S22–S209, 10.1002/alr.21695. 26889651

[6] C. Georgalas , M. Detsis , I. Geramas , D. Terzakis , and A. Liodakis , “Quality of Life Outcomes in Frontal Sinus Surgery,” Journal of Clinical Medicine 9, no. 7 (2020): 2145, 10.3390/jcm9072145. 32650386 PMC7408842

[7] P. J. Wormald , W. Hoseman , C. Callejas , et al., “The International Frontal Sinus Anatomy Classification (IFAC) and Classification of the Extent of Endoscopic Frontal Sinus Surgery (EFSS),” International Forum of Allergy & Rhinology 6, no. 7 (2016): 677–696, 10.1002/alr.21738. 26991922

[8] D. Bertossi , A. Lucchese , M. Albanese , et al., “Piezosurgery Versus Conventional Osteotomy in Orthognathic Surgery: A Paradigm Shift in Treatment,” Journal of Craniofacial Surgery 24, no. 5 (2013): 1763–1766, 10.1097/SCS.0b013e31828f1aa8. 24036775

[9] D. C. Marques , V. G. G. Pinto , R. L. de Araújo Vian , et al., “Major Approaches on the Piezoelectric Device, Drills and Saws to Orthognathic Surgery: A Systematic Review,” Health 11 (2019): 783–791, 10.4236/health.2019.116063.

[10] F. N. Bartuli , F. Luciani , F. Caddeo , et al., “Piezosurgery vs High Speed Rotary Handpiece: A Comparison Between the Two Techniques in the Impacted Third Molar Surgery,” Oral Implantology 6, no. 1 (2013): 5–10. 23991279 PMC3755811

[11] G. Mancini , S. Buonaccorsi , G. Reale , and M. Tedaldi , “Application of Piezoelectric Device in Endoscopic Sinus Surgery,” Journal of Craniofacial Surgery 23, no. 6 (2012): 1736–1740, 10.1097/SCS.0b013e318270fa16. 23147338

[12] A. Bolzoni Villaret , A. Schreiber , I. Esposito , and P. Nicolai , “Endoscopic Ultrasonic Curette‐Assisted Removal of Frontal Osteomas,” Acta Otorhinolaryngologica Italica 34, no. 3 (2014): 205–208. 24882930 PMC4035839

[13] W. E. Bolger , “Piezoelectric Surgical Device in Endoscopic Sinus Surgery: An Initial Clinical Experience,” Annals of Otology, Rhinology, and Laryngology 118, no. 9 (2009): 621–624, 10.1177/000348940911800903. 19810600

[14] V. J. Lund and I. S. Mackay , “Staging in Rhinosinusitus,” Rhinology 31, no. 4 (1993): 183–184. 8140385

[15] V. J. Lund and D. W. Kennedy , “Quantification for Staging Sinusitis. The Staging and Therapy Group,” Annals of Otology, Rhinology & Laryngology 167 (1995): 17–21. 7574265

[16] C. Hopkins , S. Gillett , R. Slack , V. J. Lund , and J. P. Browne , “Psychometric Validity of the 22‐Item Sinonasal Outcome Test,” Clinical Otolaryngology 34, no. 5 (2009): 447–454, 10.1111/j.1749-4486.2009.01995.x. 19793277

[17] S. Toma and C. Hopkins , “Stratification of SNOT‐22 Scores Into Mild, Moderate or Severe and Relationship With Other Subjective Instruments,” Rhinology 54, no. 2 (2016): 129–133, 10.4193/Rhino15.072. 27017484

[18] M. Martins , W. A. Vieira , L. R. Paranhos , et al., “Comparison of Piezosurgery and Conventional Rotary Instruments in Schneider's Membrane Sinus Lifting: A Pilot Randomized Trial,” Journal of Clinical and Experimental Dentistry 13, no. 8 (2021): e802–e808, 10.4317/jced.57953. 34512920 PMC8412807

[19] J. R. Smith , V. Patel , A. Ahmed , et al., “Comparative Analysis of Piezoelectric and Conventional Rotary Instruments in Sinus Surgery: Early Postoperative Outcomes,” Laryngoscope 128, no. 9 (2018): 2103–2110, 10.1002/lary.27145.

[20] C. H. Lee , C. C. Huang , P. W. Wu , et al., “Tissue Preservation and Thermal Safety Profile of Piezoelectric Surgery Compared With Rotary Drills in Endoscopic Sinus Procedures,” International Forum of Allergy & Rhinology 10, no. 6 (2020): 777–784, 10.1002/alr.22528.

[21] T. L. Johnson , M. Harris , and P. S. Batra , “Pain Outcomes Following Frontal Beak Reduction: A Comparison of Piezoelectric and High‐Speed Drill Techniques,” American Journal of Rhinology & Allergy 33, no. 4 (2019): 367–374, 10.1177/1945892419838657.

[22] S. Kumar , A. Dutta , and V. Ramakrishnan , “Mucosal Preservation With Piezoelectric Instruments in ESS: A Randomized Controlled Trial,” Rhinology 59, no. 3 (2021): 287–294, 10.4193/Rhin20.345.

[23] P. Martinez , P. Goyal , B. A. Senior , et al., “Piezoelectric Devices Versus Conventional Instruments in Sinus Surgery: A Systematic Review and Meta‐Analysis,” Otolaryngology‐Head and Neck Surgery 167, no. 2 (2022): 197–206, 10.1177/01945998221056749.34846979

[24] C. M. Brown , A. T. Peters , and R. R. Orlandi , “Cost Implications of Piezoelectric Instrumentation in Sinus and Skull Base Surgery,” International Forum of Allergy & Rhinology 9, no. 10 (2019): 1105–1111, 10.1002/alr.22417.31356005

[25] S. A. Nguyen , R. J. Schlosser , and Z. M. Soler , “Cost‐Benefit Analysis of Piezoelectric Versus Conventional Techniques in High‐Volume ESS Centers,” Laryngoscope Investigative Otolaryngology 5, no. 6 (2020): 1092–1098, 10.1002/lio2.508.

